# Tackling the unfolding *Anopheles stephensi* crisis in Africa: Minimise research and maximise action

**DOI:** 10.5281/zenodo.15052092

**Published:** 2025-03-19

**Authors:** Bart G.J. Knols

**Affiliations:** 1 Editor, MalariaWorld Journal

## Abstract

When the Asian tiger mosquito (*Aedes albopictus*) was discovered in the EU for the first time, in a kindergarten in Genua (Italy) in 1990, it was followed initially by a call for action to stop its spread, but gradually turned into a ‘study object’, resulting in hundreds of research papers since, but very little action in terms of actually trying to eliminate it. Europe is now facing the grave consequences of this lack of action, with dengue, Chikungunya, and West Nile virus already creating problems around the Mediterranean, and aided by climate change both the mosquito and these diseases will move farther north in years to come, posing a risk to millions of people. This history is now repeating itself in the Horn of Africa. It’s been thirteen years since *Anopheles stephensi*, an Asian malaria vector, invaded Djibouti, and has not had much going against it since. And like in Europe, *stephensi* has become ‘the new kid on the block’ for academics, and paper after paper (87 in the last 3 years) is coming online, always calling for action in the final paragraphs, but forgetting that mosquitoes don’t care about papers and happily continue to conquer more terrain as we sit down to read another paper. Research should focus exclusively on four things: where is the mosquito (surveillance), what works to kill it (control), how do we free areas of it (implementation strategy) and how can we prevent re-invasion of cleared areas. In two of these areas we’re almost clueless: strategic area-wide vector elimination and preventing re-invasion. Action in these areas is therefore critical if Africa is to prevent repeating Europe’s mistake. And should start with garnering unwavering support and resources from governments of affected countries, regional organisations, and global players. The *stephensi* problem will not be solved with a dipper searching for larvae in the field. It should start by knocking on politicians’ doors, intense lobbying, and succeeding in having the problem addressed with utmost priority. Only then will the means and resources become available to attempt elimination of this invasive vector. Critically, these resources should not affect any ongoing malaria control efforts but should be freed specifically for this purpose. On a more positive note, going aggressively after *stephensi* would concurrently teach us ways to go after indigenous African vectors in an area-wide fashion, notably through larval source management. Inertia will merely push us all decades backwards in terms of hoping to eliminate malaria in Africa one day.

In April 2024, I attended the 8th Pan-African (MIM) malaria conference in Kigali, Rwanda. There were several symposia with a focus on *Anopheles stephensi*, with presentations that focused on the genomics of this Asian vector, on molecular mechanisms of insecticide resistance, and of course worrying reports over its rapid expansion across north-east Africa, Nigeria and Ghana. Not a single one of these presentations focused on what we should do, and how we should do it, to push *stephensi* back in its tracks. As I sat through these talks, I got flashbacks of a Society of Vector Ecology (SOVE) meeting that I attended in 2003 in Bellinzona, Switzerland, where symposia focused on the alarming spread of *albopictus* in Europe. It was there that WHO’s Norman Gratz, who was working on a review of the vector status of *Aedes albopictus* at that time, which would be published a year later [1], was highly vocal and critical about the lack of action to try and eliminate *albopictus*. I was a bit shocked by his harshness, but over time had to acknowledge that he was absolutely right. Inertia angered him in the same way it angered me sitting in that conference room in Kigali. And so, at the end of the *stephensi* symposium, I asked for the microphone. And although I do not recall my exact words, I expressed my concerns about the lack of action to try and eliminate the invasion *in lieu* of more research and publications. That I feared that every next conference we would merely see new (and expanded) distribution maps of *stephensi*, and that Africa should not follow the debacle that had unfolded in Europe. There were WHO (Africa) as well as high-ranking Gates and MESA people in the audience nodding in full agreement, confirmed after the symposium during one-on-one conversations. But then everyone rushed off to the next symposium and maybe all that I had said was forgotten. Back to business as usual. Still hyped about this, I sat down at a lunch table with two young conference participants unknown to me (one from Africa, the other from the USA) that both happened to work on mosquito genomics. Neither of them had any idea about past successes in mosquito elimination and seemed oblivious to even considering this to be possible at all. Both being vector biologists, I was happy for them that Gratz wasn’t there (he passed away in 2005 [2]). We parted after both had taken note of the elimination of *Aedes aegypti* from 11 million km^2^ of South America between 1947-1962 (Europe is about 10 million km^2^), which would have likely succeeded had the programme not been terminated by US president Nixon [3], as well as the successful elimination of *Anopheles gambiae* from Brazil (during the onset of WWII).

In my book ‘Mosquito: The fascinating world of public enemy #1), which I should have written in English but was published in Dutch in 2009 [4], I elaborated extensively on the *albopictus* problem in Europe and the Netherlands in particular. Interestingly, and maybe because books (unlike research papers) are read by the general public, politicians started asking questions in the Dutch parliament about the risks and dangers posed by this and other mosquitoes. It was exciting to note some action in the political arena. Sadly, my peers, fellow medical entomologists, working for the government, that could have taken advantage of this course of action, were the very ones that pushed my warnings aside as ‘alarmist’ and ‘sensationalist’. In my book I warned that West Nile virus (transmitted primarily by *Culex* mosquitoes) would hit Europe sooner rather than later and *it did*. Ironically, the same individuals that blamed me for being alarmist contributed to an ECDC report in 2023, now ringing alarm bells themselves [5]. Too late, I’m afraid. To be more specific: Starting 3 years after my book was published, between 2012 and 2021, 16 EU/EEA countries reported a total of 3632 autochthonous cases of WNV infections in humans, with a record number of cases in 2018 (n=1551)[5]. This all makes me think of Gandhi who said:

“*Many people, especially ignorant people, want to punish you for speaking the truth, for being correct, for being you. Never apologise for being correct, or for being years ahead of your time. If you’re right and you know it, speak your mind. Even if you are a minority, of one, the truth is still the truth.*”

It was with the above in mind that I recently wrote an article about the use of chemical pesticides in malaria vector control and the risk this may bring to the environment, human health, and even food security [6]. The debate that ensued on LinkedIn following this article was ‘well, agriculture is much worse, and vector control is only using a fraction of the pesticides used in agriculture’. This is a perfect example of fallacy of relative privation ("not as bad as") – dismissing an argument or complaint due to the existence of more important problems in the world, regardless of whether those problems bear relevance to the initial argument. Big chemical corporates (e.g., Bayer, BASF, Syngenta, Sumitomo, etc.) will hate me for my opinion, but I stand up regardless. Chemical vector control is a dead-end strategy and should be replaced by more durable, and environmentally-sound (biological) control methods. Although it can be argued that judicious use of chemical pesticides is warranted when an invasive species like *stephensi* needs to be targeted, long-term use against any vector is a sure recipe for resistance and all associated problems. This sounds a bit ‘Carson-like’, but alas, I am convinced about this nevertheless.

## *Stephensi* in Africa = *Gambiae* in Brazil

In early 1930, it was likely a French marine ship that transported *Anopheles gambiae* from the harbour of Dakar, Senegal to Natal in Brazil (PCR analysis of museum specimens actually showed that these were *An. arabiensis* [7]). Strikingly, just three weeks after their arrival, larvae were detected in a pool of water by medical entomologist Raymond Shannon, who identified the invasion and raised concern. But like today, early reports of invasive mosquitoes were not considered a major threat, and priority was given to the yellow fever mosquito elimination programme [8]. And so *gambiae* spread, first along the coast, then further inland. But then, in 1938, a major malaria epidemic, likely fuelled by *gambiae,* ignited. *Plasmodium falciparum* oocyst loads on stomachs of *gambiae* were of levels never seen in African anophelines, indicating extremely high competence of the African invader to vector south-American *P. falciparum*. When this epidemic hit, Rockefeller scientists, key among them the legendary Dr. Fred L. Soper, lobbied intensively with the Brazilian government and the Rockefeller Foundation back in New York to tackle the invasion - by then it had spread over an area of 54.000 km^2^ (for your information, there are 13 African countries that are *smaller* than this area [9]). With the backing from President Vargas and (finally) large sums of money from the Foundation, Soper and a team of thousands of larval control personnel set out to treat every single possible breeding site with larvicide (mostly Paris green at that time). The story has been well documented in their book [10]. And, a mere 18 months later, they declared victory over the invasion.

I have often marvelled at this accomplishment, and together with others have argued that this could provide a nice template for African countries to tackle their malaria vectors [11]. But time and again I was whistled back by those that argued that it was ‘easy’ to eliminate *gambiae* from Brazil because it was an ‘artefact’, an African vector struggling in the dry north-east of Brazil, and was therefore an easier target than trying to target *gambiae* in its native region. And, of course, it would be argued that the elimination of *An. labranchiae*, an endemic malaria vector, had failed in Sardinia, showing that vector elimination is near-impossible for indigenous species. True or not true, with *stephensi* now in the Horn of Africa, that argument is no longer valid. Here’s an Asian vector in a new environment, this time in Africa. And so it is justifiable and worthwhile to examine how *gambiae* was removed from Brazil, learn from it, and repeat it in Africa against *stephensi*. And let’s not forget, the Brazil campaign lacked tools that we now have at our disposal that can make such a campaign much more (cost)effective, like satellite imagery, drones, GPS, mobile telephony, computers, etc. Finally, Soper was ridiculed at first when he came up with the idea to eradicate *gambiae* from Brazil. Today, we can no longer use the excuse that it would be impossible - after all, it has been done.

## From research to action

A quick search in PubMed (‘stephensi’ AND ‘Africa’; 19 March 2025) yields 149 references, with 90 of these published between 2022 and March 2025. Following the availability of funding (by organisations like the Wellcome Trust), not surprisingly, the number of papers shot up in number. The World Health Organization issued a ‘Vector alert’ in 2019 [12], and launched an initiative in 2022 to address the spread of *stephensi* in Africa through a five-pronged approach: increasing collaboration, strengthening surveillance, improving information exchange, developing guidance and prioritising research [13]. In March 2023, WHO convened a stakeholders meeting in Addis Abeba, Ethiopia [14]. Country reports were presented, research updates provided, and breakout sessions focused on control options, surveillance, etc. But today, almost two years later, concerted, big-scale action that is needed so urgently, remains fully absent. Although the President’s Malaria Initiative made its own action plan [15] - with the new US administration it is unlikely that they will be able to execute their plan. Finally, a social and behaviour change guidance document was produced in 2023 [16].

Although all of these research efforts and reports and meetings have, without doubt, good intentions, they lack plans for concerted efforts to push back the invasion and eliminate *stephensi* from areas where it has been detected. Importantly, the WHO documents nor the PMI action plan address elimination seriously. In fact, PMI opted for ‘mitigation’ of negative effects caused by *stephensi* rather than elimination. It appears that the desirable goal is complete elimination but only a half-hearted response, guaranteed to fail in the long run, is staged. Oxitec, with its 18 million dollar grant from the Gates Foundation to tackle *stephensi* in Djibouti will at best deliver a pin prick to *stephensi* populations and will ultimately fail to curb the spread and impact of this mosquito.

Research is needed, for sure, but much of what is published today has little relevance in terms of going out in the field and starting the elimination job. It is almost certain that at the time when the Brazil campaign started against *gambiae* that there were lots of unknowns about the invasive vector’s behaviour, breeding habits, response to local climate, transmission potential, insecticide susceptibility, and so on. But the battle was started regardless and the campaign learnt *on the job*. Entomological investigations on *gambiae* in Brazil took less than a year before launching a massive eradication campaign [8]. Soper, who visited South Africa in 1935, had learnt much about *gambiae* from African peers before launching the Brazil campaign. Similarly, African scientists today should build on knowledge garnered by Asian scientists when tackling *stephensi*.

By now, we know that *stephensi* in Africa can thrive in urban as well as rural settings, and that it breeds in natural as well as in man-made habitats. That means that it can be found pretty much anywhere - surveillance thus cannot be narrowed down to specific places but needs to be all-encompassing and comprehensive. Importantly, countries in which *stephensi* has not yet been found should step up surveillance efforts to detect the species as early as possible since this will increase the likelihood of successful and less costly elimination early on. *Stephensi* bites indoors and outdoors, will feed on humans and animals alike, and will lay its eggs in man-made or natural breeding sites. It will bite and feed at night but also during the daytime or at sunset [17]. Insecticide resistance has been recorded in multiple places, against different classes of recommended insecticides. Although Zhou *et al.* [17] present an impressive and solid research agenda, it can be certain that by the time all this research has been funded and conducted that the cat will be out of the bag for good and a repetition of Europe’s *albopictus* history will become a fact. The Brazil campaign started 8 years after the initial detection of *gambiae*; for *stephensi* we’ve already waited 13 years since its initial discovery in Djibouti in 2012.

By stepping up regular malaria vector control efforts, like LLINs and IRS, we’ll be able to tackle, at least partially, *stephensi* with an appetite for humans and willingness to bite and rest indoors. But for outdoor biters that feed on animals and seek shelter in animal sheds, the story becomes more complex, and for now it seems that our best bet will be to target *stephensi* in its larval stage.

However, even if that decision would be made, how does one implement a massive-scale larval source management (LSM) programme, coupled with an intensive surveillance programme? Well, if it worked in rural Brazil, then why could it not work in rural Africa? Training and deploying thousands of larval control inspectors that systematically cover terrain on a frequent basis to treat *all* breeding sites that *stephensi* could possible select. The template for such an operation is available and was published in Soper & Wilson’s book [10]. The rigour and thoroughness with which the Brazil campaign was run can be repeated, augmented with and supported by modern technology, but up front it can be said that any deviation from the goal of complete success (= elimination) will result in dismal failure.

Such a campaign, on the back of an envelope, will cost tens if not hundreds of millions of dollars. It is absolutely critical that the cost of failure or doing nothing is weighed against the massive cost of an elimination campaign. And although we could (and perhaps should) stage such a numbers exercise, the millions of additional malaria cases that will result from giving way to *stephensi* don’t make the choice very difficult - not even in front of those that have to step up to release such massive amounts of funding. For your information: The Russia-Ukraine war is estimated to cost $ 500 million to $ 1 billion **per day** [18]. Governments have money to spend - it is all about their prioritisation on what they want to spend it.

## The need for a UN special envoy for *stephensi*

Action against *stephensi* should not only be considered the realm of scientists, as it mostly appears today. We can research *stephensi* to death, only to find out later that it spread so far and wide that elimination would no longer be realistic (Europe/ *albopictus*). It is critical therefore that the highest government levels in the affected countries start to acknowledge the seriousness of this invasion. These governments, in collaboration with international bodies (like WHO, the Global Fund, etc.) can then start the vital task of bringing together the funds to get going. Lobbying is the answer to this, aided by a press that is critical about the lack of action. Politicians have many problems to manage, and it is all a matter of reaching the top of their priority list. In online meetings that I held with staff from the Global Fund, WHO and PMI, I argued that we need a UN special envoy for *stephensi*. A he or she who has the task to lobby governments, international organisations and funders, for as long as it takes until they come forward. An excellent lobbyist, not necessarily a medic or vector biologist, surrounded by a team of experts that can be called upon when needed. A charismatic, driven and passionate individual that is set on getting the job done. Previously, when the world of insecticide-treated bednets started to take off, and dreams of a net for every person in Africa came along, the UN enrolled a special envoy for malaria (Ray Chambers) who took on that role. And it helped.

No war will be started against an enemy unless the highest levels in government endorse it and provide all the manpower and resources needed to maximise the likelihood of success when going into battle. Waging a war on an invasive mosquito enemy is not much different - we can’t start it from the bottom, we should start it from the top. In Brazil, Soper got President Vargas on his side and the Rockefeller Foundation provided the funding for the campaign - two massively important steps that were vital to the success of the elimination campaign.

Five years ago, a modelling exercise published in PNAS estimated that in urban settings, if unabated, *stephensi* could pose a risk to 126 million people [19]. That’s roughly 10% of Africa’s population. If such a dazzling figure cannot drive us all into action, than what will?

## Conclusions

From the above I draw the following conclusions:

- It is imperative that the highest political echelons in countries affected by *stephensi* acknowledge that elimination is the only lasting solution and that failure to accomplish that will have massive consequences in future.
- Research funding should only be made available to gain essential knowledge to assist *stephensi* detection, methods to kill it, ways to implement those methods, and ways to prevent re-invasion. All other funding should be diverted into action on the ground.
- Leadership to tackle the *stephensi* crisis is essential. A UN special envoy for *stephensi* can take on the role of lobbying with affected countries, regional entities, and global players. Such a person will be critical in order to free resources needed to wage an eradication campaign.
- A masterplan for s*tephensi* elimination needs to be drawn up, based on historical successful vector elimination campaigns, and can be augmented with modern technology. Effective resource mobilisation and allocation should be determined on a country-by country basis and should not replace existing malaria control budgets.
- Elimination efforts should start as soon as possible, even in the face of unknowns and uncertainties. Failure is a means to learn how to become successful.
- Elimination of *stephensi* will provide major lessons for tackling indigenous malaria vectors, which, at present, still cause the bulk of malaria transmission.
- Inaction now will be sorely regretted in decades to come and will set back hopes of eliminating malaria from Africa.
